# High availability of vegetables and fruit through government-funded school lunch is not reflected in 4th grade pupils’ intake

**DOI:** 10.29219/fnr.v67.9405

**Published:** 2023-07-19

**Authors:** Cecilia Olsson, Agneta Hörnell, Maria Waling

**Affiliations:** Department of Food, Nutrition and Culinary Science, Umeå University, Umeå, Sweden

**Keywords:** school lunch, vegetables, fruit, dietary intake, pupils

## Abstract

**Background:**

An increased intake of vegetable and fruit (VF) through school meals can contribute to the prevention of non-communicable diseases.

**Objective:**

The purpose of this study was to investigate what types of VF 4th grade pupils (10–11 years old) choose, how much they eat when they are given the opportunity to serve themselves from the daily vegetable buffet available at lunch, and whether this varies with socioeconomic background and gender.

**Design:**

A cross-sectional study design was used where pupils’ VF intake was measured during 5 days with a photographic method. In total, 196 pupils from nine public schools participated.

**Results:**

The results show that pupils on average ate less than one type of VF per day from the vegetable buffet. Girls, pupils with a higher socio-economic status (SES) and those with a more frequent VF intake at home, ate more types of VF per day from the vegetable buffet than their counterparts. The median intake of VF from the vegetable buffet was generally low, 20.4 g/day. The intake was two thirds higher for pupils with higher SES in comparison with pupils with lower SES; 25 g/day versus14 g/day (*P* = 0.001). No gender differences in grams per day of VF were identified (*P* = 0.123).

**Discussion:**

This study indicates that a well-stocked vegetable buffet as part of government-funded school lunch does not automatically contribute substantially to the recommended daily intake of VF among a sample of 4th grade pupils in a high-income country like Sweden.

**Conclusions:**

The results of the study can be interpreted as a missed opportunity to increase the intentional consumption of VF among pupils in a way that would have implications for public health as well as attenuating differences between socioeconomic groups.

## Popular scientific summary

- Little is known about the impact of government-funded vegetables and fruit (VF) provision in schools. Can this increase the generally low VF intake among children in high-income countries?
- This study indicates that a VF buffet, even as part of government-funded school lunch, does not contribute substantially to the recommended VF intake among pupils aged 10–11 years in Sweden.
- Pedagogic strategies could help make VF more attractive for pupils and increase the intake.

An increased intake of vegetable and fruit (VF) is essential in the prevention of non-communicable diseases such as cardiovascular disease and some cancers (1, 2), as well as being an important part of the changes needed for sustainability (3). To prevent non-communicable diseases, the recommended population goal from the World Health Organization is to eat at least 400 g/day (4), and in Sweden the recommendation is to eat at least 500 g/day of VF. However, few children and adolescents reach that recommendation (5–8); vegetable intake is especially low (9, 10).

In a sample of 11-year-old pupils in 10 European countries, the reported VF intake was 14–45% below the World Health Organization’s recommendation (4) and the intake of vegetables was lower than for fruit (10). Sweden is no exception; the latest national dietary survey showed that less than 10% of school children reached the daily recommended intake of 500 g VF for children 10 years or older (8). The median intake per day was 152 g/day among 11-year-olds, and 172 g/day among 14-year-olds. Another recent study showed that 12-year-old Swedish pupils ate 64–65 g of vegetables during school lunch (11). Studies show that boys and children from families with low socioeconomic status (SES) consume lower amounts of VF compared to girls and children from families with higher SES (12).

Sensory aspects are other determinants for predicting VF intake in children (13). In addition to an inborn preference for sweet taste, human food preferences are learned, and the preferences developed early in life appear to be stable over time (14). Thus, a learned preference for VF early in life implies a high probability of preferring VF later in childhood and as an adult. Repeated taste exposure of a certain food is a prerequisite for preference development to take place, and availability and repeated exposure have been shown to predict the intake of VF in children and adolescents (15–17).

School meals have the potential to affect both dietary intake and future food habits among children. By reaching a large part of the population they can potentially play an important role with regard to food security among socioeconomically disadvantaged children in many parts of the world as well as in strategic health promotion interventions (18–20). Children not provided with VF at home could benefit from being served VF in school, for example, through VF programs or through government-funded meals where VF are included. A relatively recent study showed that government-funded school lunch may mitigate social inequalities in dietary intake among 11 and 14-year-old Swedish pupils (11).

A study by Bere et al. (21) showed that in schools where free fruit was provided during the school day to Norwegian pupils, the total intake of fruit increased with 72 g/day during the study period compared to a control school. Recently the same research group published a 14-year follow-up where they concluded that receiving free fruit at school for 1 year might have sustained long-term effects for less-educated women but the same was not seen for men (22). By contrast, other school-based interventions conducted to improve VF intake have had small and minimal effects on VF intake, respectively (23, 24).

The organization of school lunch varies across the world, from there being none so that pupils go home to eat, or bring food from home, to pupils being served government-funded lunches at school. Sweden is one of the few countries in the world with a long tradition of providing government-funded school lunches (25) that are also required by law to be nutritious (26). In the spring of 2013, the Swedish Food Agency published guidelines for school meals in primary schools, secondary schools and youth recreation centers (27). Those guidelines stated that a typical Swedish school lunch should consist of one or more hot dishes (usually meat or fish in the main alternative and a vegetarian option), a choice of different vegetables or salads in a buffet style (sometimes with fruit included), bread, a spreadable fat, and milk or water to drink. It was recommended that pupils should eat at least 100 g of VF in school and that a daily vegetable buffet should be served and include at least five different components (27), including at least three types of fiber rich vegetables (e.g. carrots, broccoli), at least one legume, and at least one salad vegetable (e.g. tomato, cucumber, lettuce) or fruit. The guidelines further stated that the vegetable buffet should be placed first in the serving line to promote increased intake. The guidelines have been updated in 2019 and 2021, but only with slight changes in wording (27).

Government-funded school lunch in Sweden was introduced in the 1940s with the intention of improving public health – especially among socially disadvantaged groups (25). The yearly cost of school lunch in Sweden is now approximately 6% of the total costs for the school system. In many respects, the prerequisites for Sweden as a nation to use school lunch in the promotion of increased intake of VF in the population can be viewed as optimal. Pupils are offered vegetables daily (and sometimes fruit); they are thus subjected to high availability, which hypothetically could promote the development of VF preferences and consequently increase VF intake. There are international studies showing that pupils with home-packed lunches are less likely to bring and consume VF, compared with children eating lunch served at school (28). However, few studies have been conducted regarding the contribution of government-funded school lunch on pupils’ actual dietary intake in general and more specifically the intake of VF (11).

We aim to study what types of VF pupils in 4th grade choose and how much they eat when they are given the opportunity to serve themselves from the daily vegetable buffet available at lunch, and whether this varies with socioeconomic background and gender. The focus is thus on the intake of VF that can be considered as a more active and intentional choice, rather than the intake from hot dishes where vegetables often are more invisible and therefore not necessarily consumed intentionally.

## Methods

### Study design and recruitment

This cross-sectional study was part of the Nordic school-based project entitled ‘Prospects for promoting health and performance by school meals in Nordic countries’ (ProMeal) (29). Data were collected in the school year 2013/2014, in Finland, Iceland, Norway and Sweden. However, in the present study, we only analyse data from Sweden; all pupils were in 4th grade (10–11 years old). The goal of the recruitment was to include schools from areas with diverse socioeconomic and ethnic characteristics. In the first step, schools were invited by contacting school leaders and thereafter teachers. In a second step, when a school had agreed to be part of the study, information and invitation letters together with informed consent forms were distributed to caregivers and pupils so that they could consider participation. All relevant checklist items in the STROBE-nut (30) have been followed in the reporting of the study.

### Data collection

#### Intake of vegetables and fruit during school lunch

Nine schools were visited for 5 days each. Intake of VF at school lunch was estimated with a photographic method developed and validated within the ProMeal project (31). Pupils’ meal trays at lunch were photographed by researchers and trained assistants in the school restaurants before and after intake (including any extra helpings and leftovers). Photos of the trays were taken from two angles with tripods: from above and at 45°. To link photographs to individual pupils, each pupil had a tag with an identification number that was put on the meal tray when their tray was photographed. Quantities of the separate food components on the photographed trays were estimated in grams by specially trained dietitians by comparison with reference portions with known weights; see Olafsdottir et al. (31) for more details.

The definitions of VF were based on the context in which it is traditionally used, meaning that, for example, olives and tomatoes were defined as vegetables even though both are botanically defined as fruit.

#### Intake of vegetables and fruit at home, and data on socioeconomic background

A questionnaire, developed and validated as part of the Nordic Monitoring project (32, 33), was sent out to the parents/caregivers of the pupils (i.e. parental questionnaire). The questionnaire included both questions regarding socioeconomic background (for more details, see the section on data handling and analyses below), and a food frequency questionnaire (FFQ). The FFQ covered the pupil’s food habits at home over the last 12 months whereof two questions were used in the present study. One question asked for intake of vegetables and root vegetables (vegetable juice and potato not included), and one question asked about the intake of fruit and berries (fruit juice not included). Frequency of intake was reported on a 17-level scale, from ‘none’ to ‘six or more times per day’.

### Data handling and analyses

The variables age, weight and height were normally distributed and presented as mean ± standard deviation. Age and sex specific body mass index (BMI; kg/m*^2^*) was calculated according to the International Obesity Task Force and linked to the definition of overweight at <18 years of age (BMI 25–30) and obesity (BMI ≥ 30) (34).

Total intake of VF at school was calculated as a daily mean for each pupil for the days the pupil was present in school. Intake of separate VF at school was calculated as a daily mean for each pupil for the days the pupil was present at school and the separate VF was available. On a group level, intake of VF was skewed and therefore presented as proportions (%) and median [p25:p75].

In the analysis of the proportion of pupils who ate different VF from the vegetable buffet, a new nominal variable was created. For each component, the pupil was categorized as an ‘eater’ if the pupil ate of it at least once (regardless of quantity eaten) during the 5 study days. A pupil who did not eat of an available component was categorized as a ‘non-eater’. Pupils who were not served the actual component during any of the 5 study days were categorized as ‘not served’. Pupils’ opportunity to eat a component at least once was calculated as the sum of eaters and non-eaters.

Some vegetables were served in different forms in the vegetable buffet, for example, raw carrots that were served as whole pieces, sliced or grated. When this occurred and the ‘form’ did not affect the nutrient content, vegetables were grouped together. When the ‘form’ did affect nutrient content, vegetables were separated, for example, raw cabbage pieces were separated from cabbage salad as the latter included a vinaigrette.

A total of 159 parental questionnaires included data on fruit and vegetable intake at home, which was converted to a daily median intake frequency (times per day) and treated as continuous data. The daily intake frequency was also categorized into two groups: less than two times a day, and two times a day or more.

A socioeconomic score (SES-score) reflecting the SES was created specifically for this study to facilitate analysis and decrease the number of statistical tests. The score was based on a total of five questions from the parental questionnaire: four questions covering data on educational level and occupation for both parents/caregivers, and one question on presence of money difficulties during the last 12 months (this question had four options: no money problems/had to borrow money from friends or relatives/had to ask for public assistance/been late with paying rent). The questions which the SES-score was built on were categorized into four positive factors: higher education of the respective caregiver (university degree; yes/no), the respective caregiver working outside home (yes/no), and one negative factor: money difficulties in the family (yes/no). The outcomes were then combined to produce the SES-score ranging from −1 if only the negative factor and +4 if scoring in all positive factors without the negative factor. In total, 148 parental questionnaires included complete data for both parents/caregivers to calculate an SES-score for the pupils’ families. In the statistical analysis, the SES-score was categorized in two groups: low SES < 3 and high SES ≥ 3.

Parametric and non-parametric tests were used for statistical analysis. Independent sample *t*-test was used for comparison of normally distributed variables between groups; Chi*^2^*-test was used for comparison of distributions of categorical variables, and Mann Whitney U test was used for comparison of ranks across two independent groups. Results were considered statistically significant if the two-tailed *P*-value was <0.05. Statistical analyses were conducted using IBM SPSS for Windows version 26.0.

## Results

### Participants’ background characteristics 

In total, 196 pupils from nine different schools participated in the Swedish part of the ProMeal study and provided data on school lunch intake. Included schools were run by the same municipality and located in and around a university town with 118,000 inhabitants at the time of the study.

A total of 161 caregivers (82%) answered the parental/caregiver questionnaire (Table 1); 129 (80%) of those were females. Education level of caregivers were generally high; 65% of those who filled out the FFQ and answered the question (*n* = 159), and 43% of the 2nd caregiver (*n* = 155), had at least a university degree, while 1% and 3.6%, respectively, had only 9 years of elementary school. For both caregivers, the majority were employed (90–91%). Remaining caregivers were students, on parental leave, looking for a job or on long-term sick leave (≥60 days). SES-score was calculated for 148 pupils; 62% had high SES and 38% low SES.

**Table 1:** Background information for the 196*^1^* participating pupils

Background variables	All participants	Girls (*n*= 100)	Boys (*n*= 96)	*P*
Age, years	10.5 ± 0.3*^2^*	10.5 ± 0.4	10.5 ± 0.3	0.930*^3^*
Weight, kg	37.9 ± 7.2	38.1 ± 6.6	37.6 ± 7.8	0.668*^3^*
Height, cm	145 ± 6.5	145 ± 6.7	145 ± 6.4	0.619*^3^*
BMI, kg/m^2^ *(n* = 177)	17.9 ± 2.6	18.0 ± 2.4	17.8 ± 2.8	0.695*^3^*
Prevalence of				
*Overweight (%)*4	11	13	9	0.286*^5^*
*Obesity (%)^4^*	3	1	4	
**SES-score (*n* = 145)**	3.0 [2.0; 4.0]*^6^*	3.0 [2.0; 4.0]	3.0 [2.0; 4.0]	0.605*^7^*
*University degree (%)*				
*Caregiver 1^8^ (n = 159)*	65	65	65	
*Caregiver 2 (n = 155)*	43	45	41	
*Employed (%)*				
*Caregiver 1 (n = 158)*	91	92	90	
*Caregiver 2 (n = 156)*	90	93	87	
*Money difficulties (%) (n = 158)*	6	9	3	
**Vegetable and fruit intake at home (times/day) (*n*= 159)**	2.0 [1.3; 3.0]	2.0 [1.2; 3.0]	2.0 [1.4; 3.0]	0.919*^7^*

SES-score could be between −1 and +4 and was based on five questions in the parental questionnaire covering educational level, the occupation of both caregivers, and the presence or absence of money difficulties.

1 All background data not available for all pupils, number of replies indicated when lower than 196.

2 Mean ± standard deviation (all similar numbers).

3 Independent sample *t*-test.

4 The BMI cut offs are age and sex specific and were applied for each individual according to the International Obesity Task Force and linked to the definition of overweight at 18 years of age (BMI 25–30) and obesity (BMI 30) (33).

5 Chi*^2^*-test.

6 Median [p25; p75] (all similar numbers).

7 Mann Whitney U test.

8 Caregiver 1 = the caregiver who answered the parental questionnaire and FFQ; 80% were females.

There were no statistically significant differences between girls and boys in background characteristics (Table 1). Half (51%) of the pupils were girls, and the mean age was 10.5 ± 0.3 years. Nine percent of the pupils had turned 11 years old at participation. The BMI of the group was 17.9 ± 2.6 kg/m*^2^*; 11% had overweight and 3% had obesity. Of the pupils, 77% were born in Sweden and two thirds (68%) lived fulltime with both caregivers.

The median intake frequency of VF at home was 1.0 [0.4; 1.0] and 1.0 [0.6; 2.0] times per day respectively. The median intake of VF together at home was 2.0 [1.3; 3.0] times per day (Table 1).

### Available vegetables and fruit in the vegetable buffet during school lunch 

All schools had a vegetable buffet with a content in accordance to the guidelines; however, only one of the schools had the buffet placed first in the serving line. Of the 45 main dishes served during the 5 days in the nine participating schools, 14 contained vegetables and/or legumes, of which seven were vegetarian and seven non-vegetarian. During the 38 days when the main dish was non-vegetarian, vegetarian meals were often offered as an alternative to the main dish.

Of the total 45 study days, vegetables were served in the vegetable buffet 43 days and fruit 9 days. The 2 days when vegetables were not served in the vegetable buffet, took place in two separate schools when the main meal was vegetable soup served together with fruit. Fruit was mainly served together with soup or as leftovers from a previous day with soup.

**Fig. 1:**
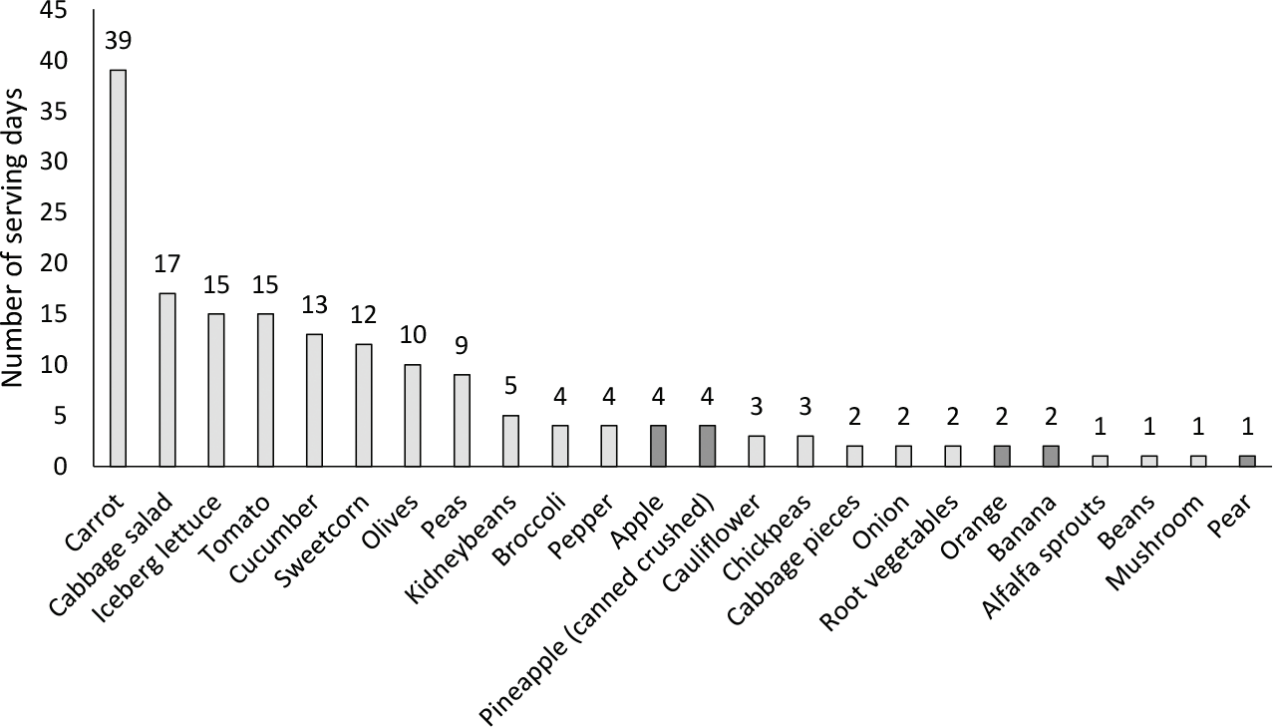
Number of serving days of VF during 45 school days representing 5 days respectively at nine schools. Bars illustrating fruits are marked in dark grey.

All schools served at least five different components of VF per day in the vegetable buffet. The most served vegetable was carrot (Fig. 1), which was served in different forms (e.g. sticks, grated, sliced), often two varieties at the same time. Out of the 45 study days, carrot was served 39 days, cabbage salad 17 days, and iceberg lettuce and tomato 15 days each. The most commonly served fruits were apple (4 days), orange and banana (2 days each).

### Pupils’ selection of available vegetables and fruit in the vegetable buffet

In total, the pupils ate less than one vegetable component per school day from the vegetable buffet; median 0.8 [0.2; 1.2] components/day (Table 2). Girls, pupils with a higher SES-score and those with a more frequent intake at home (≥2 times/day) ate more components from the vegetable buffet than boys, those with lower SES-score, and those with a less frequent intake at home (<2 times/day).

**Table 2:** Selection (number of components per day) and intake (in grams) of VF from the vegetable buffet at school lunch in relation to gender, SES-score and intake frequency at home. Numbers are presented as median [p25; p75]

Vegetable and fruit consumption	All participants *n* = 196	Gender			SES-score			Intake at home		
		Boys *n* = 96	Girls *n* = 100	*P* ^1^	Low *n* = 56	High *n* = 92	*P* ^1^	<2 times/day *n* = 72	≥2 times/day *n* = 87	*P* ^1^
Selection (no./day)										
Vegetables and fruit	1.0 [0.4; 1.5]	0.8 [0.3; 1.4]	1.2 [0.5; 1.6]	**0.013**	0.8 [0.2; 1.5]	1.1 [0.6; 1.5]	**0.034**	0.8 [0.2; 1.4]	1.1 [0.6; 1.6]	**0.011**
Vegetables	0.8 [0.2; 1.2]	0.6 [0.2; 1.0]	0.9 [0.4; 1.2]	**0.026**	0.6 [0.2; 1.0]	0.8 [0.4; 1.2]	0.105	0.5 [0.2; 1.0]	1.0 [0.4; 1.2]	**0.007**
Fruit	0.0 [0.0; 0.5]	0.0 [0.0; 0.5]	0.0 [0.0; 1.0]	0.574	0.0 [0.0; 0.5]	0.0 [0.0; 1.0]	**0.047**	0.0 [0.0; 0.5]	0.0 [0.0; 0.5]	0.315
Intake (g/day)										
Vegetables and fruit	20.4 [6.7; 36.8]	18.8 [5.6; 33.7]	22.3 [9.7; 39.3]	0.123	14.1 [3.4; 27.8]	24.5 [10.4; 39.9]	**<0.001**	14.5 [2.9; 27.3]	24.8 [11.0; 44.0]	**0.003**
Vegetables	15.4 [5.1; 30.5]	13.1 [3.7; 29.8]	16.3 [6.0; 32.1]	0.173	12.3 [3.4; 22.9]	18.9 [8.2; 31.5]	**0.027**	9.4 [2.4; 22.0]	21.0 [9.0; 37.0]	**<0.001**
Fruit	0.0 [0.0; 5.5]	0.0 [0.0; 5.0]	0.0 [0.0; 5.9]	0.654	0.0 [0.0.0]	0.0 [0.0; 8.0]	**0.029**	0.0 [0.0; 1.5]	0.0 [0.0; 5.6]	0.260

SES-score could be between −1 and +4, and was based on five questions in the parental questionnaire covering educational level, the occupation of both caregivers, and the presence or absence of money difficulties.

1 Mann Whitney U test.

**Fig. 2:**
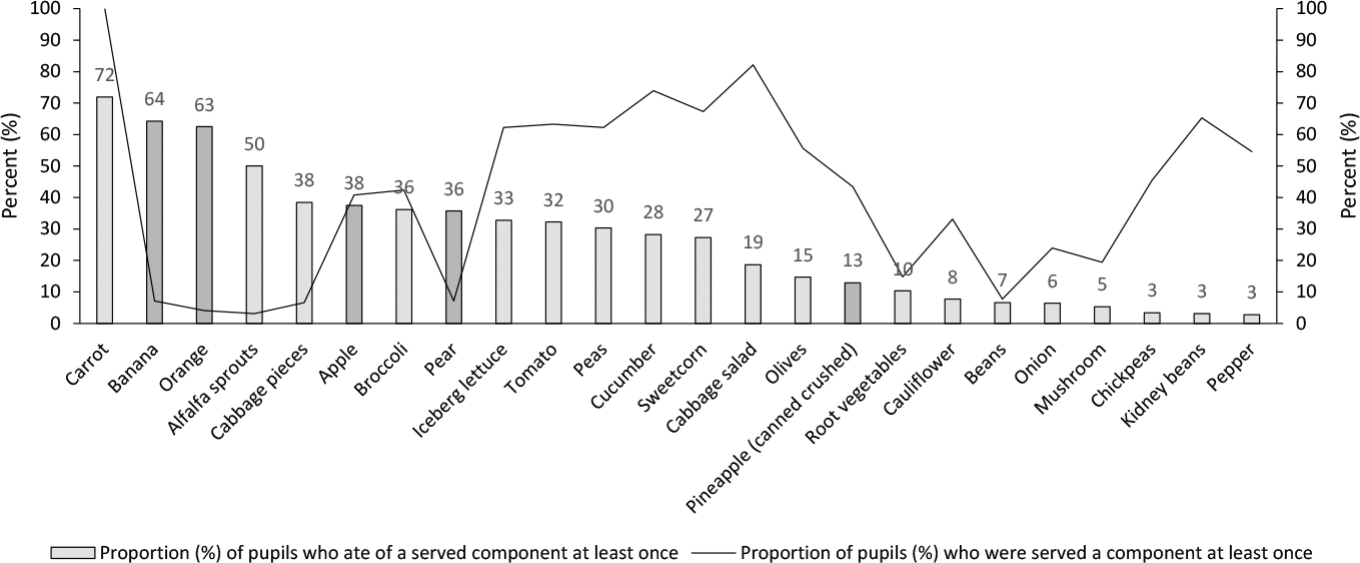
Proportion of pupils (*n* = 196) who ate different VF when it was served in the vegetable buffet. Bars illustrate the proportion of pupils who ate different VF on at least 1 day during the 5 study days when it was available in the vegetable buffet (light grey bar = vegetables; dark grey = fruit). The black line illustrates the proportion of pupils who were served the different VF on at least 1 day during the 5 study days.

Figure 2 shows the proportion of pupils with access to different VF at least once during a school week and whether the pupils ate of it when available. For most vegetables, a high proportion of pupils never ate the vegetables available to them, while fruit was popular and usually eaten by a large proportion of the pupils when they had the chance.

Carrots were available to all pupils at least once in a school week, but although it was the most popular vegetable, 28% of the pupils did not eat it. Alfalfa sprouts was the second most popular vegetable and eaten by 50% of the pupils when they had the chance; however, it was only served once in one school. Cabbage pieces, broccoli, iceberg lettuce, tomato, peas, cucumber and sweetcorn were eaten by about one third of the pupils (27–38%), and the proportion of pupils who ate other vegetables varied between 3 and 19%.

The proportion of pupils served fruit at least once was generally low at 4–7% for most types of fruit. Apple was an exception as it was served to 41% of the pupils. When fruits were available, it was usually popular among the pupils and most fruits were eaten by 36–64% of the pupils. Canned crushed pineapple was the least popular fruit and only 13% of the pupils ate it, despite it being available to almost half of the pupils at least once, mainly mixed with cabbage.

### Pupils’ intake of vegetable and fruit during school lunch 

Four pupils (2%) had an average VF intake from the vegetable buffet during the 5 studied days that met the planned intake of 100 g/day, while 22 pupils (11%) ate no vegetables or fruit at all from the vegetable buffet over the 5 days.

The median intake of VF was low at 20.4 [6.7; 36.8] g per day (Table 2). The intake was two thirds higher for pupils with high SES-score and those with higher intake frequency at home (twice per day or more), compared with those with low SES and those with a lower intake than twice per day, respectively. There were no gender differences in the median intake of VF combined, nor taken separately (*P* = 0.123 – 0.654).

**Fig. 3:**
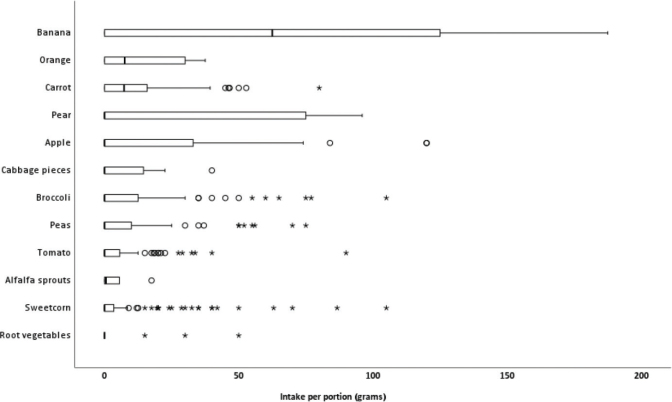
Pupils´ median daily intake of separate VF served in the vegetable buffet. The boxes are limited to the 12 components where pupils had the highest median intake. The vertical black bold line denotes the median value (50th percentile) while the box contains the 25th and 75th percentiles. ‘o’ depicts outliers, and ‘*’ depicts extreme values.

The vegetables that primarily contributed to the overall vegetable intake in grams were carrot, cabbage pieces, broccoli, and peas (Fig. 3). Fruit was usually served halved or whole, resulting in larger portion sizes, especially for banana with a median intake of 62.5 [0.0; 125] g/portion.

## Discussion

One of the more outstanding results of this study was that the 10–11 years old pupils consumed what can be considered as extremely low quantities of vegetables from the vegetable buffet despite a variety being available every day. Furthermore, one of 10 pupils did not eat vegetables or fruit from the buffet at all. It is obvious that the pupils’ low consumption (a total median of 20.4 g [6.7; 36.8] g/day) during school lunch in this study did not contribute substantially to reach either the 100 g of VF per pupil that many schools use for planning amounts in the vegetable buffet, or the Swedish daily recommendation of 500 g vegetables, fruit, and berries from 10 years of age (27).

In general, the nine included schools adhered to the national guidelines (27) of serving a warm meal together with a daily vegetable buffet including at least five different components (vegetables, mixed vegetables, or fruit). Yet, the median intake in the group was less than one vegetable component per pupil and day from the vegetable buffet. Furthermore, in this relatively homogenous group from a highly educated background, our results confirm the link between SES and VF intake seen in many studies (35), as pupils with a higher SES-score had a slightly higher median intake of VF from the vegetable buffet in comparison with pupils with a lower SES-score. Whether the relatively small differences (about 11 g for fruits and vegetables, and only 7 g for vegetables) have any practical health implications may, however, be questioned.

The vegetable intake during school lunch (15.4 [5.1; 30.5] g per day) found in the present study was considerably lower than in a Swedish study performed during the school year 2016/2017 on a national representative sample in grades 5 (9 years old) and 8 (14 years old) (11). That study showed that the pupils in grade 5, close in age to the pupils in the present study, consumed 64–65 g of vegetables during school lunch. Part of the difference is most likely due to the fact that the study by Eustachio Colombo et al. included estimated amounts of vegetables from both the vegetable buffet and the main dishes, whereas the present study only included vegetables from the vegetable buffet. Furthermore, the national guidelines regarding placement of the vegetable bar first in the serving line to promote increased VF intake (27), had been in place for 3 years in the study by Eustachio Colombo et al. (11), while it was recently launched at the time of the present study and not yet adhered to in all participating schools. In addition, it is possible that the general opinion about vegetarian meals had also become more positive in the intermediate years. There were also important differences in methodology between the national study and the present one. In the present study, researchers and trained dietitians estimated amounts on the pupils’ plates from photographs taken during 5 days, including both taken food and leftovers ensuring a high precision in the estimates. By contrast, Eustachio Colombo et al. (11) used 24-h-recall for 3 separate days, and the pupils had to estimate their own VF intake in retrospect using pictures with standard portion sizes. Fewer days were also included in the assessment (i.e. 1–3 days). It cannot be ruled out that the high reported intake of VF in Eustachio Colombo et al. study was partly due to overestimation caused by the methodological factors mentioned.

It is well known that children from families with lower SES have a less healthy diet and eat lower quantities of VF at home compared to families with a higher SES both in the Nordic countries (36), and elsewhere (37, 38). Schools in Sweden have a stated mission to compensate for differences in pupils’ SES-background with the aim of improving their chances of performing well in school (39). Regardless of their opportunities for eating healthy food at home, children always have the opportunity of eating a nutritious meal during school days. However, when focusing on the vegetable buffet in school specifically, our results indicate that the vegetable buffet does not contribute substantially to the planned compensatory contribution since children with higher SES-scores and those who had a higher intake frequency of VF at home, had a slightly higher median intake and ate more components from the vegetable buffet during school lunch than their counterparts. So, although the vegetable buffet contributed to an increased total intake for all and made pupils get closer to the recommended intake of VF, a gap between the groups remained. In contrast to Eustachio Colombo et al. (11), this indicates that a government-funded school lunch system providing VF does not necessarily mean that it reduces intake differences between socioeconomic groups in a high-income country like Sweden.

The question is whether it is reasonable to expect that overall well-nourished pupils should eat a lot of VF simply because it is available. We have previously shown that pupils experience stress and a lack of time during school lunch (40), something that can partly explain the low intake of VF in the present study. The placement of the vegetable buffet after the pupils had taken their main meal was also a likely contributing factor to the low intake because it would leave less space on the plate for the VF-components. Also, if pupils are hungry and have limited time for eating, it is likely that visual cues of the served meal components support the decision to choose more energy-dense components of the meal (41), filling the plate already before reaching the vegetable buffet. This can be especially true if taste preferences of low energy-dense foods such as vegetables are not developed among pupils. Taste preferences have been shown as a main reason for pupils not to eat VF (16, 42), making them less likely to save space for these components. Taste preferences are linked to how familiar pupils are with different tastes, that is, development of taste preferences presupposes repeated exposure, not only visually, but also through taste experiences (15 – 17). While fruits usually are sweet and easily accepted, many vegetables have bitter tastes and also harder textures requiring more frequent exposure before acceptance (43). The present study indicates that repeated visual exposure to a wide range of VF during school lunch does not seem to be sufficient to make a significant contribution to the overall intake of fruit and vegetables in 10–11-year-old-children. The intention behind the guidelines to serve at least five different vegetable components during school lunch is good but schools` adherence to the guidelines will merely contribute to visual exposure if the pupils do not taste the vegetables.

Our results indicate the importance of finding new knowledge on how to pedagogically promote taste exposure of vegetables, and thereby increase the likelihood of an adequate intake among children. Pedagogic meals are common in Sweden (26), but there is little scientific evidence regarding the extent to which these contribute to promoting healthy eating habits among pupils and how best pedagogic meals should be designed and organized. Integration of school lunch in the educational activities in school is highlighted in a cross-national review (44). The possibility of having ‘fruit breaks’, which also could include raw vegetables, either before or after lunch should be considered as a way to increase overall VF intake, but also as an easy way to get a better distribution of food intake during the day. At present, Sweden is only taking part of the milk in the European Union school fruit, vegetables and milk scheme (45), and there is a possibility to also utilize the fruit and vegetable part. The scheme economically supports distribution of fruit, vegetables and milk to school children in the European Union and comes with educational measures to teach children about healthy eating habits. Taking part of the full scheme would enable Swedish schools to serve pupils additional fruit and vegetables without an extra cost, and it could also support the integration of educational activities related to VF.

In our study, several types of vegetables were served almost every school day; however, the consumed amounts were low. One approach to increase the overall intake of vegetables among the pupils could be a shift from serving five different components in the vegetable buffet, to serve only one or two basic components that pupils tend to like (e.g. carrots and cabbage pieces). To broaden the taste preferences of vegetables, this base can be complemented with alternating exposure to other vegetables, one by one, as repeated exposure is required for taste preference to develop. This could preferably take place within the framework of interdisciplinary teaching, especially when there is limited time to perform planned pedagogic activities during school lunch. Although, challenges such as practical difficulties with scheduling and the lack of time have been reported in relation to interdisciplinary teaching, the advantages are increased learning opportunities for pupils (46).

In our study, pupils ate relatively large amounts of fruit when these were served. However, fruit was served less often than vegetables and usually only when soup was the main meal. Based on our results, it is likely that the pupils would have eaten a larger total amount of VF if fruit had been served more frequently. In the national guideline (27), fruit are included in the same group as ‘salad vegetables’ (e.g. cucumber, tomato, iceberg lettuce) and the advice is that at least one of the salad vegetables should be served per day. Fruit could therefore have been served more frequently as one of the components in the vegetable buffet (or in fruit breaks as mentioned above). The reason why the participating schools chose not to serve fruit daily could be that fruit is generally more expensive than vegetables and limitations in the national production caused by the geographical situation. Berries are however included as an important food in the Nordic Nutrition Recommendations (47). Increased use of domestic berries, for example, blueberries and lingonberries in schools could be a valuable, sustainable addition to fruits and vegetables in the Swedish context. However, due to the seasonal availability of domestic fruit (also certain vegetables) in Sweden, increased serving of fruit in school could interfere with the climate goal included in current national guidelines to reduce food miles (48). Our suggestion to include fewer varieties of vegetables in the buffet, with a base of vegetables that pupils actually eat (carrot, cabbage) is therefore also favorable from a sustainability perspective. Those vegetables are also storable and can be easily included in hot dishes. Another effect of mainly serving vegetables that pupils actually eat is that it could contribute to less waste from the buffet (49).

### Methods discussion

An advantage of the present study is the detailed photographic dietary assessment method used to assess VF intake from the vegetable buffet during school lunch. As opposed to many previous studies where amounts of VF have been assessed either with pictures of portion sizes or standard portions, the pupils in the present study took their VF as usual and researchers and trained dietitians approximated the amounts from pictures of each individual pupil’s plate. Also, the photographic method was previously validated against weighed records showing that the agreement between estimated energy content in the school lunch was very close to the true measurement (31). Furthermore, 5 days of assessment were included for most of the pupils making it more probable to catch the pupils’ habitual eating pattern of VF than fewer study days.

As in all dietary assessments there are numerous measurement errors involved and it is not possible to rule out that the pupils changed their food intake during the study days and made more healthy choices. However, our impression was that the pupils ate as usual. In addition, the 5 days of data collection made them accustomed to having the research team around. Furthermore, it is not possible to rule out that the validated FFQ used to assess intake at home was biased. Well known limitations related to FFQ are the difficulty of remembering what has been consumed, fixed food lists that do not include what the participants have consumed, and that parents who fill out FFQ for their children may have difficulties in knowing what the child has consumed.

As mentioned earlier, the intention was to recruit schools from a variety of socioeconomic areas, but as the municipality included a university town with a generally high socioeconomic population, we did not succeed in getting the large variation in socioeconomic areas aimed for. This means that the results of this study may not represent other parts of Sweden, or places where there is a larger variation in SES. It should further be noted that the data collection in the present study was performed in 2013/2014 and it is not possible to rule out that the dietary intake of VF from the salad bars in schools has changed since then.

## Conclusions

This study indicates that a well-stocked vegetable buffet as part of government-funded school lunch does not automatically contribute substantially to the recommended daily intake of VF among a sample of 4th grade pupils in a high-income country like Sweden. The nutritious school meal system in Sweden is costly but constitutes an easy way to reach all children regardless of background and is important for public health equality. The results of the study can be interpreted as a missed opportunity to increase the intentional intake of VF among pupils in a way that would have implications on public health as well as attenuating differences between socioeconomic groups. Future research should therefore focus on how VF can be made more attractive for pupils to eat at school and how pedagogic strategies can contribute to an increased and expanded intake.
